# Supplementary material for: Extracellular electron transfer increases fermentation in lactic acid bacteria via a hybrid metabolism
Source: eLife. 2022 Feb 11;11:e70684. doi: 10.7554/eLife.70684 (PMC8837199; doi:10.7554/eLife.70684)
Supplement: Supplementary file 1. [file elife-70684-supp1.docx]

# Supplementary file 1. Data used for calculating the bioenergetic balances.

|  | **Mannitol consumed** | **Lactic acid** | **Acetic acid** | **Ethanol** | **Succinic acid** | **Pyruvic acid** | **Formic acid** | **Dry weight** | **Electrons to anode** |
| --- | --- | --- | --- | --- | --- | --- | --- | --- | --- |
|  | mM | mM | mM | mM | mM | mM | mM | g/L | mM |
| EET (anode) | 10.05 ± 2.58 | 12.41 ± 0.33 | 0.55 ± 0.13 | 2.12 ± 1.38 | 0.021 ± 0.006 | 0.86 ± 0.16 | 0.07 ± 0.01 | 0.052 ± 0.04 | 12.87 ± 0.96 |
| OCP | 5.28±1.5 | 3.46 ± 0.4 | 0.12 ± 0.04 | 1.01 ± 0.49 | 0.01 ± 0.00 | 0.83 ± 0.15 | 0.09 ± 0.08 | 0.046 ± 0.009 | - |

The following standards were included in the HPLC measurements: acetate, formate, pyruvate, malate, lactate, succinate, oxalacetate, fumarate, ethanol, acetoin, butanediol, mannitol and glucose. No gaseous products were measured.

# Carbon recovery-EET conditions (fermentation products+biomass): 79±5 %

# Carbon recovery-OC conditions (fermentation products+biomass): 55±5 %

A 53% of the biomass was considered to be C, as previously reported (Shuler and Kargi, 2002)

**Methods to calculations of NADH regeneration**

The concentration of NADH regenerated is calculated as the difference between the NADH consumed and produced through EET and the formation of fermentation products. Calculating the concentration of NADH consumed is straightforward. We assume NADH is consumed as a result of both fermentative pathways associated with substrate-level phosphorylation and EET pathways. For the substrate-level phosphorylation pathways, we assume that 1 mol of lactate, ethanol, or succinate each consume 1, 2 or 2 mol of NADH, respectively. For EET pathways, the concentration of NADH consumed was calculated by first determining the moles of electrons harvested by the anode:

$mol electrons=charge (A*s)* Faraday constant (96485 \frac{mol e}{A*s})$ Eqn 1

where

$charge=current (A)*time (s)=current density (\frac{A}{cm^{2}})* area anode (cm^{2}) * time (s)$ Eqn 2

Then, the concentration of NADH regenerated was calculated using the volume of the reactor and the fact that 2 electrons are released in the oxidation of NADH to NAD^+^ basis:

$[NADH]=mol electrons*2 \frac{mole NADH}{mol electrons}*\frac{1}{0.15 L reactor}$ Eqn 3

Because the amount of mannitol consumed cannot be completely accounted for by biomass and fermentation products, there are two different assumptions that can be made to calculate the NADH produced. These two assumptions represent lower and upper bounds on the concentration of NADH regenerated. Using these bounds, we provide a range of NADH concentrations in Table 1.

**Method 1: Establishing a lower bound of NADH regenerated**

This method assumes that the production or consumption of NADH is only due to formation of fermentation products. Since 79% of the carbon in consumed mannitol was recovered in fermentation products and biomass under EET conditions, this method accounts for the majority of the NADH produced. However, this method does not account for any undetected metabolites and may underestimate the total NADH produced. Thus, we consider this calculation to be a lower bound on the amount of NADH regenerated.

This method uses the known stoichiometry of the fermentation pathways (McFeeters and Chen, 1986) to assume 1.5 mol of NADH is produced for each mole of lactate, acetate, ethanol, pyruvate, succinate and formate detected. Using this method, we estimate that 96% of the NADH is reoxidized under EET conditions. Of this fraction, 63% of the NADH is reoxidized by substrate-level phosphorylation and 27% by EET. In contrast, under OC condition, 69% of the NADH produced is reoxidized via substrate-level phosphorylation.

**Method 2: Establishing an Upper Bound of NADH regenerated**

This method assumes NADH production is directly related to disappearance of mannitol. Thus, this method overestimates this fraction since a proportion of this mannitol will be used for biomass synthesis and will not lead to NADH generation.

This method uses the known stoichiometry of mannitol oxidation to assume that 3 mol of NADH is produced for each mole of mannitol that is consumed. Calculations performed using this method estimate that 77% of the NADH is reoxidized under EET conditions. Of this fraction, 55% of the NADH is regenerated through substrate-level phosphorylation and 21% via EET. In contrast, 66% of the NADH produced is reoxidized via substrate-level phosphorylation under OC conditions.

**References**

Shuler M.L., Kargi F. 2002. Bioprocess Engineering: Basic Concepts. Prentice Hall.
