# Supplementary material for: Extracellular electron transfer increases fermentation in lactic acid bacteria via a hybrid metabolism
Source: eLife. 2022 Feb 11;11:e70684. doi: 10.7554/eLife.70684 (PMC8837199; doi:10.7554/eLife.70684)
Supplement: Supplementary file 2. [file elife-70684-supp2.docx]

# Supplementary file 2. Comparison of the energy metabolism discovered in this study with fermentation in LAB and anaerobic respiration in *Geobacter* spp.

|  | **Homofermentation in LAB** | **Respiration in LAB** | **Anaerobic EET respiration in *Geobacter* spp.** | **Hybrid metabolism in LAB (this study)** |
| --- | --- | --- | --- | --- |
| Reduction of insoluble extracellular electron acceptor | No | No | Yes  4-8 mA/mg protein at peak  (Marsili et al., 2010; Rose and Regan, 2015) | Both - soluble and insoluble  1.5 mA/mg- protein  (at peak**^a^**) |
| NADH:quinone oxidoreductase is required for reduction of electron acceptor | No (Tachon et al., 2010) | Ndh1 (Brooijmans et al., 2009b, 2009b) | Yes  Ndh1 (proton pumping) (Chan et al., 2017) | Yes  Ndh2 (non-proton pumping) |
| Electron acceptor transcriptionally upregulates  NADH dehydrogenase | No (this study) | No [aerobic respiration] (Pedersen et al., 2008) | Yes (Holmes et al., 2006) | Yes (with DHNA) |
| NAD^+^/NADH ratio | ~1 for glucose (Guo et al., 2017, p. 201)  ~5 for mannitol (this study) | Near-zero for lactose (Johanson et al., 2020) | 10 (Fe^3+^)  10-100 (anode) (Rose and Regan, 2015; Song et al., 2016) | 260 (Fe^3+^)  45 (anode) |
| Fraction of electrons on electron acceptor | 40-98% pyruvate (Dirar and Collins, 1972) | 33% (nitrate) (Brooijmans et al., 2009, p. 1) | 75-90% anode (Speers and Reguera, 2012) | 20% anode  48% pyruvate |
| Metabolic flux is primarily through | Fermentative pathway (Bintsis, 2018) | Mixed-acid fermentation [(Brooijmans et al., 2009a)](https://www.zotero.org/google-docs/?kpfm2o) | TCA cycle (Galushko and Schink, 2000; Mahadevan et al., 2006) | Fermentative pathway - Mixed acid |
| ATP yield per substrate | ~2-3 mol ATP/mol glucose for homolactic (Dirar and Collins, 1972)  0.75 mol ATP/mol mannitol (this study) | 3.3-3.9 mol ATP/mol lactose (Johanson et al., 2020) | ~1.5 mol ATP/mol substrate**^b^** (Mahadevan et al., 2006) | 1.6 mol ATP/mol mannitol |

**^a^** Calculated assuming that 50% of the dry cell weight is protein. ^b^ *G. sulfurreducens* uses acetate as its electron donor. Since acetate is a 2 carbon electron donor, ATP yield is expressed per mol of a 6 carbon substrate.

**References**

Bintsis, T., 2018. Lactic acid bacteria as starter cultures: An update in their metabolism and genetics. AIMS Microbiol. 4, 665–684. https://doi.org/10.3934/microbiol.2018.4.665

Brooijmans, R., de Vos, W.M., Hugenholtz, J., 2009a. Electron transport chains of lactic acid bacteria - walking on crutches is part of their lifestyle. F1000 Biol. Rep. 1. https://doi.org/10.3410/B1-34

Brooijmans, R., Smit, B., Santos, F., van Riel, J., de Vos, W.M., Hugenholtz, J., 2009b. Heme and menaquinone induced electron transport in lactic acid bacteria. Microb. Cell Factories 8, 28. https://doi.org/10.1186/1475-2859-8-28

Brooijmans, R.J.W., Vos, W.M. de, Hugenholtz, J., 2009. *Lactobacillus plantarum* WCFS1 electron transport chains. Appl. Environ. Microbiol. 75, 3580–3585. https://doi.org/10.1128/AEM.00147-09

Chan, C.H., Levar, C.E., Jiménez-Otero, F., Bond, D.R., 2017. Genome scale mutational analysis of *Geobacter sulfurreducens* reveals distinct molecular mechanisms for respiration and sensing of poised electrodes versus Fe(III) oxides. J. Bacteriol. 199. https://doi.org/10.1128/JB.00340-17

Dirar, H., Collins, E.B., 1972. End-products, fermentation balances and molar growth yields of homofermentative lactobacilli. J. Gen. Microbiol. 73, 233–238. https://doi.org/10.1099/00221287-73-2-233

Galushko, A.S., Schink, B., 2000. Oxidation of acetate through reactions of the citric acid cycle by *Geobacter sulfurreducens* in pure culture and in syntrophic coculture. Arch. Microbiol. 174, 314–321. https://doi.org/10.1007/s002030000208

Guo, Y., Tian, X., Huang, R., Tao, X., Shah, N.P., Wei, H., Wan, C., 2017. A physiological comparative study of acid tolerance of *Lactobacillus plantarum* ZDY 2013 and L. plantarum ATCC 8014 at membrane and cytoplasm levels. Ann. Microbiol. 67, 669–677. https://doi.org/10.1007/s13213-017-1295-x

Holmes, D.E., Chaudhuri, S.K., Nevin, K.P., Mehta, T., Methé, B.A., Liu, A., Ward, J.E., Woodard, T.L., Webster, J., Lovley, D.R., 2006. Microarray and genetic analysis of electron transfer to electrodes in *Geobacter sulfurreducens*. Environ. Microbiol. 8, 1805–1815. https://doi.org/10.1111/j.1462-2920.2006.01065.x

Johanson, A., Goel, A., Olsson, L., Franzén, C.J., 2020. Respiratory physiology of *Lactococcus lactis* in chemostat cultures and its effect on cellular robustness in frozen and freeze-dried starter cultures. Appl. Environ. Microbiol. 86. https://doi.org/10.1128/AEM.02785-19

Mahadevan, R., Bond, D.R., Butler, J.E., Esteve-Nuñez, A., Coppi, M.V., Palsson, B.O., Schilling, C.H., Lovley, D.R., 2006. Characterization of metabolism in the Fe(III)-reducing organism *Geobacter sulfurreducens* by constraint-based modeling. Appl. Environ. Microbiol. 72, 1558–1568. https://doi.org/10.1128/AEM.72.2.1558-1568.2006

Marsili, E., Sun, J., Bond, D.R., 2010. Voltammetry and growth physiology of *Geobacter sulfurreducens* biofilms as a function of growth stage and imposed electrode potential. Electroanalysis 22, 865–874. https://doi.org/10.1002/elan.200800007

Pedersen, M.B., Garrigues, C., Tuphile, K., Brun, C., Vido, K., Bennedsen, M., Møllgaard, H., Gaudu, P., Gruss, A., 2008. Impact of aeration and heme-activated respiration on *Lactococcus lactis* gene expression: identification of a heme-responsive operon. J. Bacteriol. 190, 4903–4911. https://doi.org/10.1128/JB.00447-08

Rose, N.D., Regan, J.M., 2015. Changes in phosphorylation of adenosine phosphate and redox state of nicotinamide-adenine dinucleotide (phosphate) in *Geobacter sulfurreducens* in response to electron acceptor and anode potential variation. Bioelectrochemistry Amst. Neth. 106, 213–220. <https://doi.org/10.1016/j.bioelechem.2015.03.003>

Speers, A.M., Reguera, G., 2012. Electron donors supporting growth and electroactivity of *Geobacter sulfurreducens* anode biofilms. Appl. Environ. Microbiol. 78, 437–444. https://doi.org/10.1128/AEM.06782-11
