# Supplementary material for: Extracellular electron transfer increases fermentation in lactic acid bacteria via a hybrid metabolism
Source: eLife. 2022 Feb 11;11:e70684. doi: 10.7554/eLife.70684 (PMC8837199; doi:10.7554/eLife.70684)
Supplement: Supplementary file 3. [file elife-70684-supp3.docx]

**Supplementary file 3. Strains and plasmids used in this study.**

| **Strains** | **Isolation Source / Description** | **Reference** |
| --- | --- | --- |
| *L. plantarum* NCIMB8826 | Human saliva | (Dandekar, 2019) |
| *L. plantarum* NCIMB8826-R | Rifampicin-resistant mutant of NCIMB8826 | (Yin et al., 2018) |
| *L. plantarum* MLES100 | Deletion mutant of NCIMB8826 lacking *ndh2* | This study |
| *L. plantarum* MLES101 | Deletion mutant of NCIMB8826 lacking *pplA* | This study |
| *L. plantarum* MLEY100 | Deletion mutant of NCIMB8826 lacking *narGHIJ* | This study |
| *L. plantarum* B1.3 | Brown flour teff injera | (Yu et al., 2021) |
| *L. plantarum* AJ11 | Fermented olives; commercial fermentation | (Yu et al., 2021) |
| *L. plantarum* 8.1 | Wheat boza | (Yu et al., 2021) |
| *L. plantarum* ATCC 202195 | Human stool | (Wright et al., 2020) |
| *L. plantarum* NCIMB700965 | Cheese | (Heeney and Marco, 2019) |
| *L. pentosus* BGM48 | Fermented olives | (Golomb et al., 2013) |
| *L. casei* BL23 | Dairy products | (Mazé et al., 2010) |
| *L. brevis* ATCC 367 | Silage | (Makarova et al., 2006) |
| *L. lactis* KF147 | Mung bean sprouts | (Siezen et al., 2010) |
| *L. lactis* IL1403 | Cheese | (Bolotin et al., 2001) |
| *L. rhamnosus* GG | Human intestine | (Kankainen et al., 2009) |
| *L. murinus* ASF361 | Mice | (Wannemuehler et al., 2014) |
| *E. faecalis* ATCC 29212 | Urine | (Minogue et al., 2014) |
| *E. faecium* ATCC 8459 | Cheese | (Kopit et al., 2014) |
| *P. pentosaceus* ATCC 25745 | Plants | (Makarova et al., 2006) |
| *S. agalactiae* ATCC 27956 | Bovine udder infection | (McDonald and McDonald, 1976) |
| *E. coli* DH5α | *fhuA2 lac(del)U169 phoA glnV44 Φ80' lacZ(del) M15 gyrA96 recA1 relA1 endA1 thi−1 hsdR17,*  amplification of cloning vector | (Taylor et al., 1993) |
| **Plasmids** |  |  |
| pRV300 | EryR AmpR, *E. coli* Ori pMB1, integrative vector | (Leloup et al., 1997) |
| pRV300:ndh2 | pRV300 derivative used for deletion of *ndh2* | This study |
| pRV300:pplA | pRV300 derivative used for deletion of *pplA* | This study |
| pRV300:narG | pRV300 derivative used for deletion of *narG* | This study |
