# Supplementary material for: Extracellular electron transfer increases fermentation in lactic acid bacteria via a hybrid metabolism
Source: eLife. 2022 Feb 11;11:e70684. doi: 10.7554/eLife.70684 (PMC8837199; doi:10.7554/eLife.70684)
Supplement: Supplementary file 4. [file elife-70684-supp4.docx]

**Supplementary file 4. Chemically defined medium.**

| **CDM component ^a^** | **Final concentration (g/L)** |
| --- | --- |
| **Buffers and salts** |  |
| MOPS (3-(N-morpholino)propanesulfonic acid) | 8.371 |
| K_2_HPO_4_ | 0.871 |
| NH_4_Cl | 1.070 |
| Na_2_SO_4_ | 1.420 |
| **Metals** |  |
| MgCl_2_ * 6H_2_O | 0.203 |
| MnCl_2_ * 4H_2_O | 0.001 |
| FeSO_4_ * 7H_2_O | 0.014 |
| **Amino acids** |  |
| Casamino acids | 3.000 |
| Cysteine-HCl * H_2_O | 0.145 |
| Tryptophan | 0.050 |
| **Wolfe’s Vitamins^b^** |  |
| Pyridoxine HCl | 0.002 |
| Thiamine HCl | 0.001 |
| Riboflavin | 0.001 |
| Nicotinic acid | 0.001 |
| Calcium D-(+)-pantothenate | 0.001 |
| *p*-Aminobenzoic acid | 0.001 |
| Thioctic acid (α-Lipoic acid) | 0.001 |
| Biotin | 0.0004 |
| Folic acid | 0.0004 |
| Vitamin B12 | 0.00002 |
| **Wolfe’s Minerals^c^** |  |
| Nitrilotriacetic acid (NTA) | 0.3 |
| MgSO_4_ * 7H_2_O | 0.6 |
| MnSO_4_ * H_2_O | 0.1 |
| NaCl | 0.2 |
| FeSO_4_ * 7H_2_O | 0.02 |
| CoCl_2_ * 6H_2_O | 0.02 |
| CaCl_2_ | 0.02 |
| ZnSO_4_ * 7H_2_O | 0.02 |
| CuSO_4_ * 5H_2_O | 0.002 |
| AlK(SO)_4_ * 12H_2_O | 0.002 |
| H_2_BO_3_ | 0.002 |
| Na_2_MoO_4_ * 2H_2_O | 0.002 |

**^a^** All solutions were prepared separately and sterile filtered through a 0.22 μm filter before combining. Glucose or mannitol was also supplemented at 22.520 g/L or 22.772 g/L, respectively.

**^b^** pH adjusted to 11 before sterile filtering.

**^c^** After NTA addition, pH adjusted to 8 before adding remaining components and sterile filtering.
