# Supplementary material for: Extracellular electron transfer increases fermentation in lactic acid bacteria via a hybrid metabolism
Source: eLife. 2022 Feb 11;11:e70684. doi: 10.7554/eLife.70684 (PMC8837199; doi:10.7554/eLife.70684)
Supplement: Supplementary file 5. [file elife-70684-supp5.docx]

**Supplementary file 5. Primers developed for this study.**

| **Primer ID** | **Sequence^a^** | **Description** | **Use** |
| --- | --- | --- | --- |
| *ndh2*-A | CCG**GAATTC**GGCGGACACATACTTGGTC | Upstream of *ndh2* | Deletion construction (EcoRI cut site) |
| *ndh2*-B | *GAAGCAGCTCCAGCCTACAC*TCTCATTACGACGGTGTTAAAAC | Upstream of *ndh2* | Deletion construction (SOEing PCR overlap region) |
| *ndh2*-C | *GTGTAGGCTGGAGCTGCTTC*GCATCGAGGTTTGAACGGAA | Downstream of *ndh2* | Deletion construction (SOEing PCR overlap region) |
| *ndh2*-D | GGG**GAGCTC**TGCCGTTCTTGTTCACTTGG | Downstream of *ndh2* | Deletion construction (SacI cut site) |
| *ndh2*-Check-F | TCGTGGCCTTAATTTCAACC | Upstream of *ndh2* deletion | Confirmation of gene deletion |
| *ndh2*-Check-R | CCGGCGTTTTGTAATTGTTCC | Downstream of *ndh2* deletion | Confirmation of gene deletion |
| *pplA*-A | TAAGCA**GAATTC**CCGTTCGGTAGCAACTTCAT | Upstream of *pplA* | Deletion construction (EcoRI cut site) |
| *pplA*-B | *GAAGCAGCTCCAGCCTACAC*TCCCACTCCTAACCTTTTTGT | Upstream of *pplA* | Deletion construction (SOEing PCR overlap region) |
| *pplA*-C | *GTGTAGGCTGGAGCTGCTTC*GCTGAGGGCCTTTTGTTTTT | Downstream of *pplA* | Deletion construction (SOEing PCR overlap region) |
| *pplA*-D | TAAGCA**GAGCTC**TTAAACGCCCCAGCTAACAC | Downstream of *pplA* | Deletion construction (SacI cut site) |
| *pplA*-Check-F | TTGCGTAAACACCAGCAAAC | Upstream of *pplA* deletion | Confirmation of gene deletion |
| *pplA*-Check-R | GCTGCTTTGATCATTGGGTA | Downstream of *pplA* deletion | Confirmation of gene deletion |
| *narG*-A | GCGAAA**GTCGAC**AAGCAGCCAGTCAGTAATAG | Upstream of *narG* | Deletion construction (SalI cut site) |
| *narG*-B | *GAAGCAGCTCCAGCCTACA*CTCACCGATAAGACCTCCTTT | Upstream of *narG* | Deletion construction (SOEing PCR overlap region) |
| *narG*-C | *GAAGCAGCTCCAGCCTACA*GCACAAATTGGGATGGTCTT | Downstream of *narG* | Deletion construction (SOEing PCR overlap region) |
| *narG*-D | GCGAAA**CCGCGG**CATCACGCTTATACATCGCC | Downstream of *narG* | Deletion construction (SacII cut site) |
| *narG*-Check-F | TACATTGCGTTAGGACCGAA | Upstream of *narG* | Confirmation of gene deletion |
| *narG*-Check-R | CCATAACCACCGACCATTTG | Downstream of *narG* | Confirmation of gene deletion |
| *rpoB*-F | CGATGACTCTAACCGTGC | *rpoB* | RT-PCR |
| *rpoB*-R | CAAGGCAATCCCTGAGTC | *rpoB* | RT-PCR |
| *ndh2*-F | CCGGCTGTCCAGATTAACGT | *ndh2* | RT-PCR |
| *ndh2*-R | CGAAGCAGCGCCGACTATTA | *ndh2* | RT-PCR |
| *pplA*-F | GCTCTGCCGCTACTGGTAAC | *pplA* | RT-PCR |
| *pplA*-R | TCGCACCAGTCACAACATCA | *pplA* | RT-PCR |

**^a^** Restriction sites for enzymes are in bold, SOEing PCR overlap sequences are italicized.
