# Supplementary material for: Extracellular electron transfer increases fermentation in lactic acid bacteria via a hybrid metabolism
Source: eLife. 2022 Feb 11;11:e70684. doi: 10.7554/eLife.70684 (PMC8837199; doi:10.7554/eLife.70684)
Supplement: Supplementary file 6. [file elife-70684-supp6.docx]

**Supplementary file 6. Transcriptome read counts, alignment rate, and gene assignment rate.**

| **Sample ID** | **Total paired reads** | **Paired reads passed quality filtering^a^** | **Reads passed quality control (%)** | **Reads aligned to *Lp*^b^** | **Alignment (%)** | **Assigned reads^c^** | **Read assignment rate (%)** | **rRNA/**  **tRNA/ncRNA Reads** | **Remaining reads for DE^d^** | **Remaining reads for DE^d^ (%)** |
| --- | --- | --- | --- | --- | --- | --- | --- | --- | --- | --- |
| mMRS-1 | 3.91E+07 | 3.40E+07 | 86.8 | 3.38E+07 | 99.6 | 30287036 | 89.6 | 10670771 | 19616265 | 64.8 |
| mMRS-2 | 3.96E+07 | 3.46E+07 | 87.2 | 3.44E+07 | 99.5 | 29762763 | 86.6 | 11736601 | 18026162 | 60.6 |
| mMRS-3 | 3.43E+07 | 3.03E+07 | 88.4 | 3.02E+07 | 99.5 | 26518101 | 87.8 | 13822383 | 12695718 | 47.9 |
| DHNA-1 | 4.13E+07 | 3.59E+07 | 87.0 | 3.57E+07 | 99.5 | 31800634 | 89.0 | 11364096 | 20436538 | 64.3 |
| DHNA-2 | 3.83E+07 | 3.35E+07 | 87.5 | 3.33E+07 | 99.5 | 29672126 | 89.0 | 9933102 | 19739024 | 66.5 |
| DHNA-3 | 3.99E+07 | 3.49E+07 | 87.5 | 3.47E+07 | 99.6 | 30954603 | 89.1 | 14806368 | 16148235 | 52.2 |
| Iron-1 | 3.31E+07 | 2.88E+07 | 86.8 | 2.86E+07 | 99.4 | 25147375 | 87.9 | 7180253 | 17967122 | 71.4 |
| Iron-2 | 2.47E+07 | 2.18E+07 | 88.2 | 2.16E+07 | 99.4 | 18943484 | 87.5 | 4976026 | 13967458 | 73.7 |
| Iron-3 | 3.10E+07 | 2.67E+07 | 86.1 | 2.66E+07 | 99.7 | 23221100 | 87.4 | 7577947 | 15643153 | 67.4 |
| Both-1 | 3.66E+07 | 3.03E+07 | 82.6 | 3.01E+07 | 99.6 | 26811334 | 88.9 | 12603853 | 14207481 | 53.0 |
| Both-2 | 4.39E+07 | 3.86E+07 | 87.9 | 3.85E+07 | 99.7 | 34163533 | 88.8 | 16841230 | 17322303 | 50.7 |
| Both-3 | 3.58E+07 | 3.11E+07 | 86.9 | 3.10E+07 | 99.7 | 27593814 | 89.0 | 14617870 | 12975944 | 47.0 |

**^a^** Paired-end reads filtered through Trimmomatic (ver. 0.39)

**^b^** Paired-end reads aligned to *L. plantarum* genome with Bowtie2 (ver. 2.3.5)

**^c^** Reads assigned to *L. plantarum* genes with FeatureCounts (ver. 1.6.4)

**^d^** Differential expression analyses done through DEseq2 on DEBrowser (ver. 1.14)
